# Transcriptional profiling of *Microtus fortis* responses to *S. japonicum*: New sight into Mf‐Hsp90*α* resistance mechanism

**DOI:** 10.1111/pim.12842

**Published:** 2021-06-09

**Authors:** Dehui Xiong, Saiqun Luo, Kunlu Wu, Yuanjing Yu, Jiameng Sun, Yanpeng Wang, Jingping Hu, Weixin Hu

**Affiliations:** ^1^ Molecular Biology Research Center School of Life Science Central South University Changsha China; ^2^ Department of Laboratory Animal Central South University Changsha China

**Keywords:** HSP90*α*, IL‐10‐JAK2/STAT1, *Microtus fortis*, RNA‐Seq, *Schistosoma japonicum*

## Abstract

**Aims:**

Schistosomiasis is a parasitic disease with a chronic debilitating character caused by parasitic flatworms of the genus *Schistosoma*. The main disease‐causing species of *Schistosoma* in China is *S. japonicum*. *M fortis* has been proved to be a nonpermissive host of *S. japonicum*. Mf‐HSP90*α* (*Microtus fortis* heat shock protein 90alpha), the homologue of HSP90*α*, display anti‐schistosome effect in vitro and in vivo. In the current study, in order to investigate the mechanism of anti‐schistosome effect of Mf‐HSP90*α*, we conducted RNA‐Seq to obtain the transcriptome profile of *M. fortis* liver infected with *S. japonicum* at different time points.

**Methods and Results:**

By mapping the differential expressed genes (DEGs) to Gene Ontology (GO) and Kyoto Encyclopedia of Genes and Genomes (KEGG), we found that the JAK2/STAT1 pathway was highly enriched with an elevated level of IL‐10 and HSP90*α*. We then checked the IL‐10‐JAK2/STAT1‐HSP90*α* pathway, and found that this pathway was activated in the infected mice with *S. japonicum*. The expression of the molecules in this pathway was elevated on the 10^th^ day after infection and gradually decreased on the 20^th^ day.

**Conclusions:**

The IL‐10‐JAK2/STAT1‐HSP90*α* axis was associated with the anti‐schistosome effect of Mf‐HSP90*α*, and targeting IL‐10‐JAK2/STAT1‐HSP90*α* axis might be a novel therapeutic strategy for developing resistance to *S. japonicum* infection.

## 1 INTRODUCTION

Schistosomiasis is a tropical disease that is caused by parasitic flatworms of the genus *Schistosoma*.^1^ Schistosome infection is caused by penetration of cercatiae.^2^ Adult male and female worm mate and produce fertilized eggs in veins of their hosts. The eggs partly lodge in the intestines or liver within the host, where they are able to induce granulomatous host immune response.^3^ The granuloma formation further induces chronic inflammation which leads to clinical manifestations such as abdominal pain, diarrhoea and rectal bleeding.^4^ The *Schistosoma* species that can infect humans have distinct geographical distributions. Among them, *Schistosoma japonicum* is the most common disease‐causing species in China and the Philippines.^5^ Early effective treatment needs to be carried out in order to eliminate the threat of *Schistosoma* infection on human health.

*Microtus fortis* is one of the rodents living on the shores of the Dongting Lake, Hunan province, China, where *S. japonicum* is highly epidemic. *M. fortis* has been proved to be a nonpermissive host of *S. japonicum*.^6^ *S. japonicum* starts to grow slowly, become atrophied and finally died at 3‐4 weeks after infection in *M. fortis*. The sera of *M. ​fortis* are found to have anti‐schistosome effects in vivo by passive immunization.^7^, ^8^ Moreover, many studies have been conducted using *M. fortis* to investigate therapeutic strategies against schistosomiasis caused by *S. japonicum*.^9^, ^10^, ^11^, ^12^, ^13^, ^14^

We have conducted a series of studies to unveil the mechanism of extermination effect of *M. ​fortis*. Firstly, we found that the extermination effect on schistosomula of the sera of *M. fortis* was stronger than that of mice, but no significant difference in anti‐schistosomula effect was found in vitro between tissue and organ of *M. fortis*.^15^ Then, we compared killing effect of different fractional proteins from *M. fortis* serum to *S. japonicum* juveniles, and we found the serum albumin and Karyopherin alpha 2 (KPNA2) of. *M. fortis* have natural resistance to *S. japonicum* infection.^16^, ^17^, ^18^ Moreover, we screened a *M. fortis* marrow cDNA library by expression cloning, the conditioned medium of full‐length of Mf‐HSP90*α* showed anti‐schistosome function in vitro. The mice transferred with Mf‐HSP90*α* displayed higher reduction in worm burden and liver eggs, indicating anti‐schistosome ability of Mf‐HSP90*α* in vivo.^19^ However, the exact mechanism of anti‐schistosome effect of Mf‐HSP90*α* was still unclear. Therefore, this study was designed to further investigate the mechanism of anti‐schistosome effect of Mf‐HSP90*α*.

## 2 MATERIALS AND METHODS

### 2.1 Animals and parasites

Sexually mature closed colonies of *M. fortis*, provided by Department of Laboratory Animal, Central South University, Changsha, China, were used in this study. All animals were acclimatized for a week prior to the experiment. The infected *Oncomelania hupensis* snails were obtained from Hunan institute of Parasite Disease, Yueyang, China. We carried out all the animal experiments in strict accordance with the Laboratory Animal Regulation (1988.11.1) and made every effort to minimize suffering. All the procedures related to the use of experimental animals had been approved by the Ethical Committee of School of Life Science, Central South University (License No.2017‐2‐5). We monitored the health of the animals daily. Liver issues were harvested after animals were euthanized by isoflurane exposure, frozen in liquid nitrogen, and stored at −80°C for the RNA and protein extraction. These animals were housed in groups of three per cage with free access to food and water on a 12 h light/dark cycle. Every effort was made to minimize the suffering of animals.

### 2.2 Animal infection and sample collection

The infected *Oncomelania hupensis* snails were placed in dechlorinated water under artificial light to induce cercarial releasing prior to infection. Eighteen *M. fortis* were randomly divided into three groups including uninfected group (control group), infected for 10 days group, infected for 20 days group, with 6 *M*. *fortis* in each group. *M. fortis* were cutaneous infected with *S. japonicum* cercariae. *M. fortis* was infected with 1000 cercariae per individual. After infected with *S. japonicum* for 10 days (n = 6) and 20 days (n = 6), *M. fortis* were sacrificed, respectively. Six *M. fortis* were used as uninfected controls. We randomly selected three per group for liver transcriptome sequencing (three biological duplications).

### 2.3 Transcriptome sequencing and data assembly

Transcriptome sequencing was performed by Novogen Biotechnology Co., Ltd. Total RNA was isolated using TRIzol reagents according to the manufacturer's instructions. RNA concentration was measured using Qubit® RNA Assay Kit in Qubit® 2.0 Flurometer (Life Technologies). RNA integrity was assessed using the RNA Nano 6000 Assay Kit of the Agilent Bioanalyzer 2100 system (Agilent Technologies). mRNA was purified from total RNA using poly‐T oligo‐attached magnetic beads. A total amount of 3 µg RNA per sample was used as input material for the RNA sample preparations. Sequencing libraries were generated using NEBNext® Ultra™ RNA Library Prep Kit for Illumina® (NEB) following the Illumina protocol and sequenced on an Illumina HiSeq™ 2000 platform. Transcriptome assembly was accomplished by using Trinity.^20^

### 2.4 Bioimformatics analysis of transcriptome

Differential expression analysis of two samples was performed using the DEGseq (2010) R package. *P*‐value was adjusted using Benjamini and Hochberg's approach. ^21^ Adjusted *P*‐value <.05 was set as the threshold for significantly differential expression. GO enrichment analysis of the differential expressed genes (DEGs) was implemented by the GOseq R packages based Wallenius noncentral hypergeometric distribution.^22^ KEGG enrichment analysis of the DEGs was accomplished by the KOBAS software.^23^ The sequences of the DEGs was blast (blastx) to the genome of a related species in the STRING database (http://string‐db.org/) to get the predicted protein‐protein interaction (PPI) relationship of these DEGs. To obtain an overview of the comprehensive information of the gene functions, we annotated the unigenes in seven major databases: NCBI nonredundant protein sequences (NR), NCBI nonredundant nucleotide sequences (NT), KEGG Ortholog database (KO), Swiss‐Prot protein databases (SwissProt), Protein family (Pfam), Gene Ontology (GO) and Clusters of eukaryotic Orthologous Groups of proteins (KOG).

### 2.5 Reagent and antibodies

The Western Blotting Substrate Kit (32132) was purchased from Thermo Fisher Scientific™. The bicinchoninic acid (BCA) protein assay kit (23227) was purchased from Pierce™. The TRIzol reagents were purchased from Life Technologies. The Revert Aid Reverse Transcriptase was purchased from Thermo Fisher Scientific™. The TB Green™ Premix Ex Taq™ was purchased from Takara. anti‐IL‐10 (Cat. abs128784), anti‐JAK2 (Cat. abs118049), anti‐STAT1 (Cat. abs115001), anti‐Hsp90 (Cat. abs130560a), anti‐phospho‐JAK2 (Cat. abs139986a) and anti‐phospho‐STAT1 (Cat. abs130924a) were purchased from Absin Bioscience Inc. anti β‐Actin(Cat. CW0096) was purchased from Beijing Comwin Biotech Co., Ltd.

### 2.6 Real‐time PCR

Total RNA was isolated using TRIzol reagents according to the manufacturer's instructions. Complementary DNA synthesis was carried out using Revert Aid Reverse Transcriptase. TB Green™ Premix Ex Taq™ was used for qPCR. The primers used for gene expression analysis were listed in Table 1. Briefly, Trizol reagent (Invitrogen Inc,) was utilized to separate the total RNA. Then, 2 µg total RNA was reversely transcribed to cDNA. After that, cDNA was applied to conduct qRT‐PCR. PCR amplification process was, initially desaturating at 95℃ for 30 seconds, 40 cycling at 95℃ for 5 seconds, annealing at 60℃ for 30 seconds, and subsequently extending for 30 seconds. mRNA expression was standardized as β‐actin, the internal inference. 2^−ΔΔCt^ method was applied for the final results. Ct value refers to the cycle number when fluorescent signals reach the threshold.

**TABLE 1:** The primers used for gene expression analysis

Primer name	forward (5′to 3′)	reverse (5′to 3′)
OAS1A	AGAGATGCTTCCGAGACA	ACTGGCGAGATTGTTAAGG
OAS2	GCCAGAGCAAGCCTTATG	ATCTTCCAGTTCCTCATCCT
DSRAD/ADAR1	GCTGCGTAGAGAAGTAGG	CTCCGTCTTCACTGTTCAA
MX1	GAAATCGCTGCCACTACT	TCTCATTGCCGTCTTCTG
HSP70	CTATTCTGAGTGGTGATAAGTC	GCTGGTTGTCTGAGTAAGT
ORF2	GTGTGGGCATAACTGGATA	TTCTGTTGAGGTCTCTGATC
DEPP	GAGAAGAGATGCCAGGTC	TCTCCTCTCAGTCTGTTGT
HMGB3	CGTGGACTTCATCGCTTA	AGAGGATTAGGAACATCACC
IL10	ATGCTCTTACTGGCTGGA	TCGGTAAGCAGTATGTTG
JAK2	CGCAGATTCATTCAGCAGTTC	GTGTAGAAGGCAGACTGTAGG
STAT1	TCTGGTTCCCTGCCTTCTTT	CTGTTGGAAGAGCACGAAGG
HSP90a	GCCTCTGGAGATGAGATGGT	AGCCGAATTAGCAACTTGGT
β‐actin	AACAGTCCGCCTAGAAGCAC	CGTTGACATCCGTAAAGACC

### 2.7 Western blot analysis

Liver tissues were homogenized and lysed in RIPA assay buffer. Proteins (25‐35 µg) were separated using 12.5% sodium dodecyl sulphate/polyacrylamide gel electrophoresis and then transferred onto polyvinylidene fluoride membranes (Millipore). After blocking, membranes were incubated with appropriate dilutions of primary antibodies, horseradish peroxidase‐conjugated secondary antibodies, respectively, and visualized using the ECL system.

### 2.8 Statistical analysis

All experiments were performed for at least three times. All statistical analyses were carried out using SPSS 19.0. The data values were presented as the mean ± standard deviation. Differences in mean values between two groups were analysed by two‐tailed t test, and the mean values of more than two groups were compared with one‐way analysis of variance. Significant differences were shown by an asterisk (*P < .05, ** *P* < .01, *** *P* < .001).

## 3 RESULTS

### 3.1 Illumina sequencing and data assembly

In order to analyse the expression profiles of the DEGs in the liver of *M. ​fortis*, we performed the comparative transcriptome to analyse the resistance‐related genes. It was reported that massive haemorrhage in the lungs, vacuolar degeneration in the hepatocytes and dilated liver sinusoids were the major pathophysiological changes observed in *M. fortis* in 6‐10 days after infected with *S. japonicum*, and these changes would gradually be recovered from the 20^th^ day after infection.^24^ After removing adaptors and low‐quality reads, we generated 57 521 645, 51 117 613 and 56 575 685 clean reads from each group. We then assembled 465,846 transcripts (mean length: 410 nt) and 371 251 unigenes (mean length: 343 nt), respectively, with Trinity software. High‐quality, long sequences enabled us to collect more information on relevant genes (Figure S1, Tables 2,3).

**TABLE 2:** Summary of the sequencing data quality for each group

Parameters	Uninfected	Infected for 10 d	Infected for 20 d
raw reads NO.(n)	59 390 019	52 912 985	58 459 771
Clean reads NO.(n)	57 521 645	51 117 613	56 575 685
clean bases(bp)	7.19	6.39	7.07
Q30 percentage (%)	91.59	90.86	91.02
GC percentage (%)	47.73	48.89	48.58

**TABLE 3:** Summary of the assembly statistics

Assembly description	Numbers
Total transcripts (n)	465 846
Number of unigenes(n)	371 251
N50 of transcripts (bp)	2284
N50 of unigenes (bp)	880
Median Length of transcritps (bp)	410
Median Length of unigenes (bp)	343

### 3.2 Annotation and classification of reference transcriptome

**FIGURE 1:**
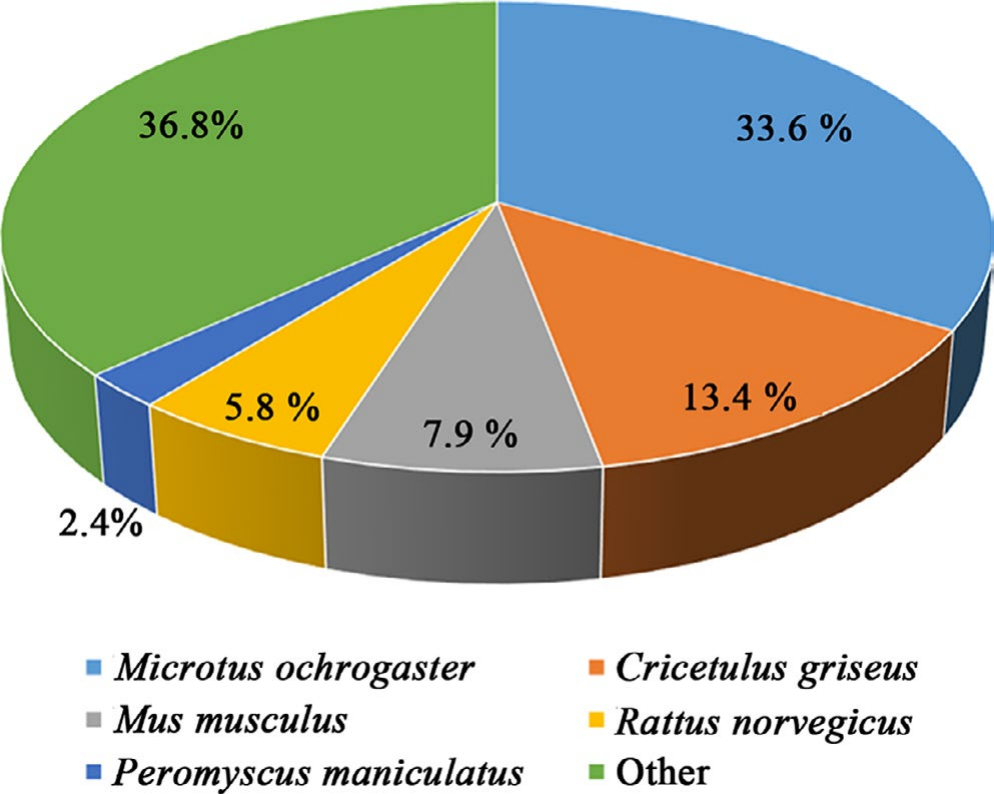
Top‐hit species distribution for sequences submitted BLASTX against the NR database of NCBI

Approximately 34.78% (129 154 out of 371 251) unigenes were significantly matched with those in one or more databases, while 2.51% unigenes were annotated by all databases (Table 4). To obtain the similarity between the gene sequence of *M. fortis* and that of related species, we compared the unigenes of *M. fortis* within the NR database. For most sequences in NR, similar unigenes were retrieved from *Microtus ochrogaster* (14 212, 33.6%), *Cricetulus griseus* (5 644, 13.4%), *Mus musculus* (3 359, 7.9%), *Rattus norvegicus* (2 453, 5.8%) and other species (21 602, 29.4%) (Figure 1).

**TABLE 4:** The annotation statistics

Annotated in Database	Number of Unigenes	Percentage (%)
NR	42 293	11.39
NT	111 368	29.99
KO	18 239	4.91
SwissProt	31 992	8.61
Pfam	40 756	10.97
GO	41 162	11.08
KOG	15 185	4.09
in all databases	9326	2.51
at least one database	129 154	34.78
Total Unigenes	371 251	100

Abbreviations: GO, Gene Ontology; KO, KEGG Ortholog database; KOG, Clusters of euKaryotic Orthologous Groups of proteins; NR, NCBI nonredundant protein sequences; NT, NCBI nonredundant nucleotide sequences; Pfam, Protein family; SwissProt, A manually annotated and reviewed protein sequence database.

**FIGURE 2:**
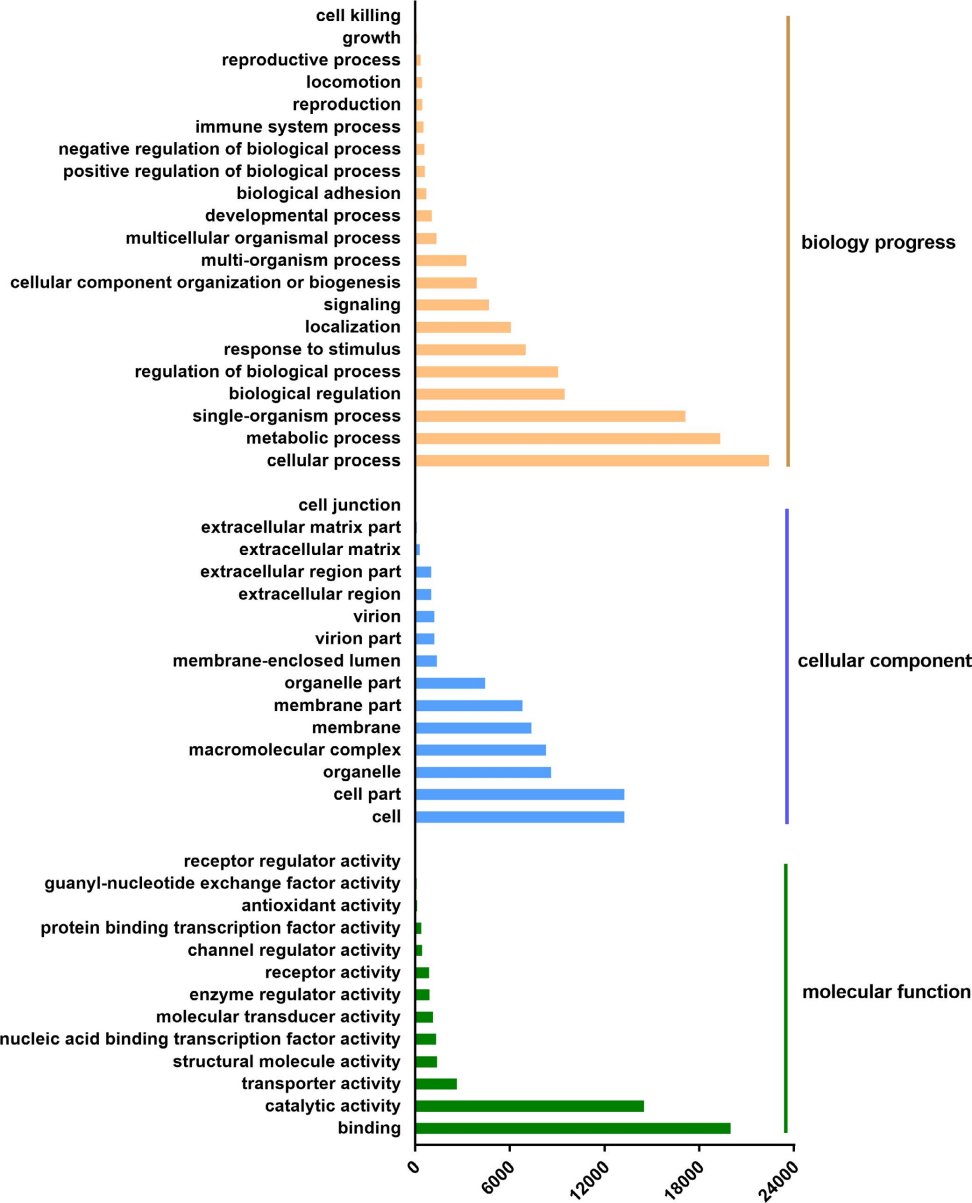
The GO annotation of assembled unigenes. GO functional classification (level2) of the annotated 371 251 unigenes. GO terms listed above were significantly enriched GO terms with correct *P*‐value <.05. X‐axis, terms with numbers of unigenes in the major category. Y‐axis, three major functional categories of GO terms: biological process, cellular component and molecular function

**FIGURE 3:**
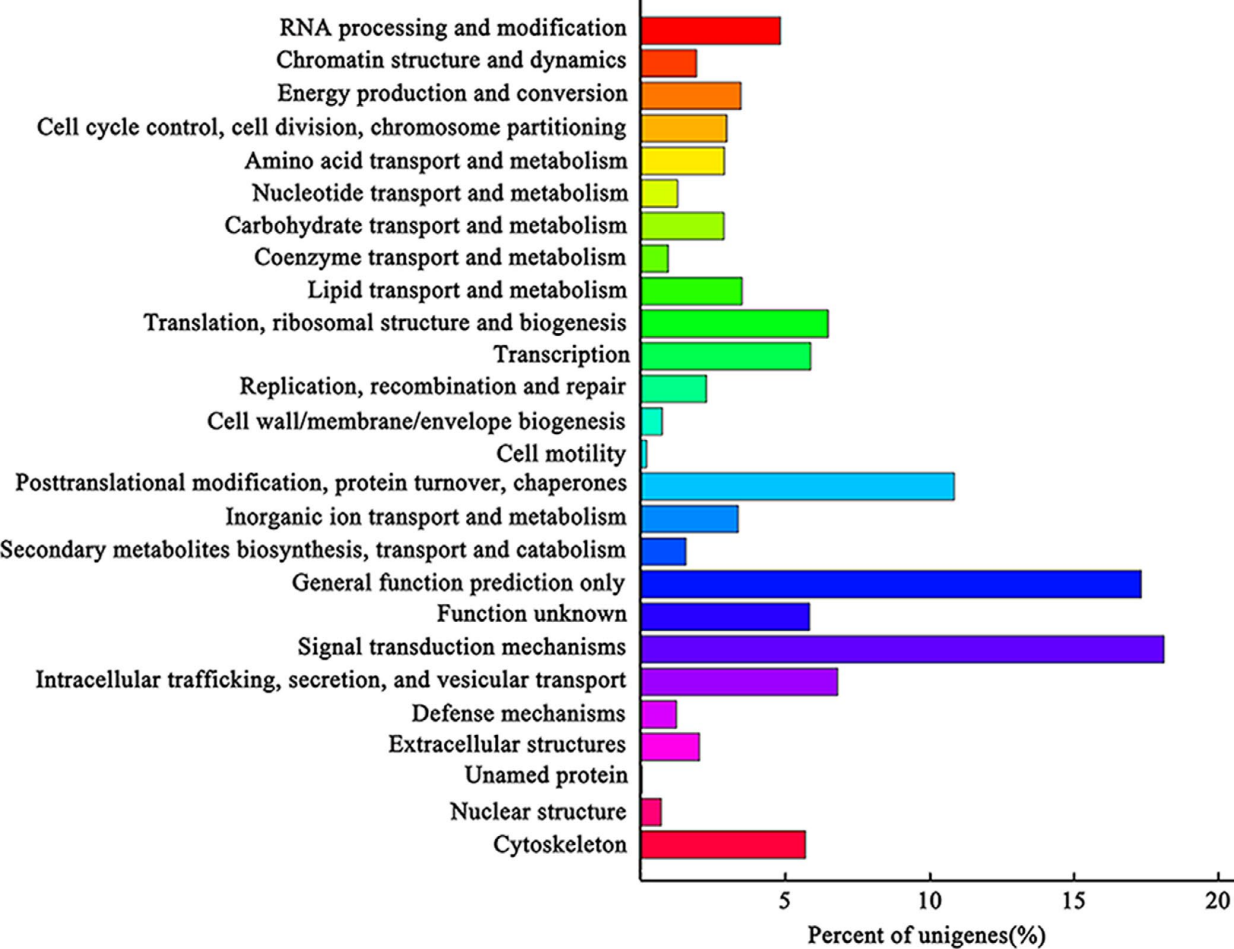
The KOG annotation of assembled unigenes. X‐axis, percentage of unigene annotated in the group; Y‐axis, the name of 26 groups in KOG

**FIGURE 4:**
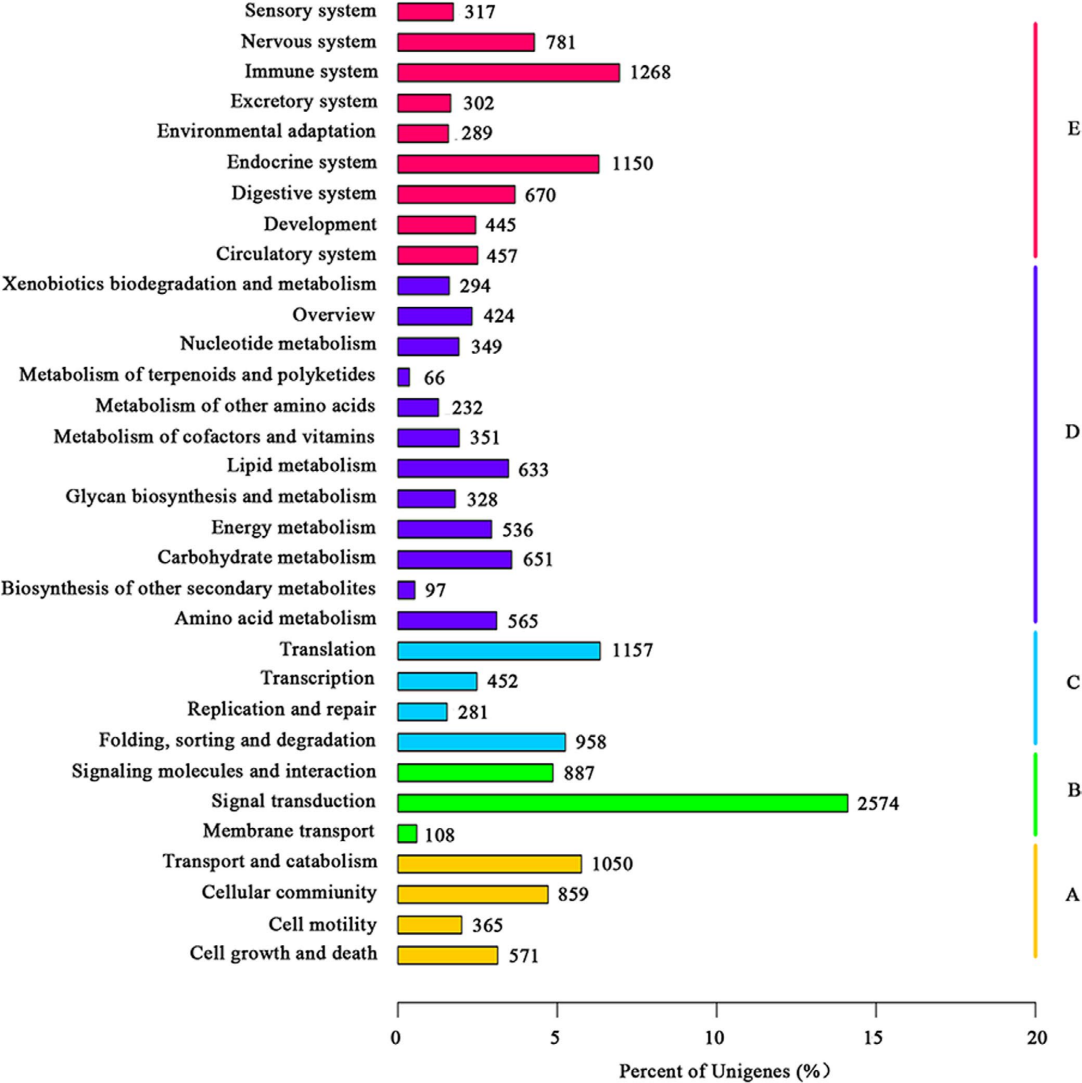
The KEGG annotation of assembled unigenes. Five capital letters with the corresponding coloured bars indicate five main categories. (A) cellular processes; (B) environmental information processing; (C) genetic information processing; (D) metabolism; (E) organism systems

We then utilized GO analysis to categorize 41 162 annotated genes into three groups according to their functions, including biological process, cellular component and molecular function (Figure 2). In the first group, 19 332, 7 016 and 534 unigenes were enriched in metabolic process, response to stimulus and immune system, respectively, (Table S1). Additionally, results from the KOG functional analysis categorized 15 185 unigenes (11.8%) into 26 groups (Figure 3). Among them, the largest group was the ‘Signal transduction mechanism’ group (2 749 unigenes, 18.10%), followed by the ‘General function prediction only’ group (2 629 unigenes, 17.31%) and the ‘Posttranslational modification, protein turnover, chaperones’ group (1 646 unigenes, 10.84%) (Figure 3 and Table S2). Moreover, results from the KEGG analysis indicated that the top five enriched pathways in ‘organismal systems’ were ‘Immune system’ (1 268, 6.95%), ‘Endocrine system’ (1 150, 6.31%), ‘Nervous system’ (781, 4.28%), ‘Digestive system’ (670, 3.67%) and ‘Circulatory system’ (457, 2.51%) (Figure 4 and Table S3).

### 3.3 DEGs expression patterns between control and infected groups at different time points

**FIGURE 5:**
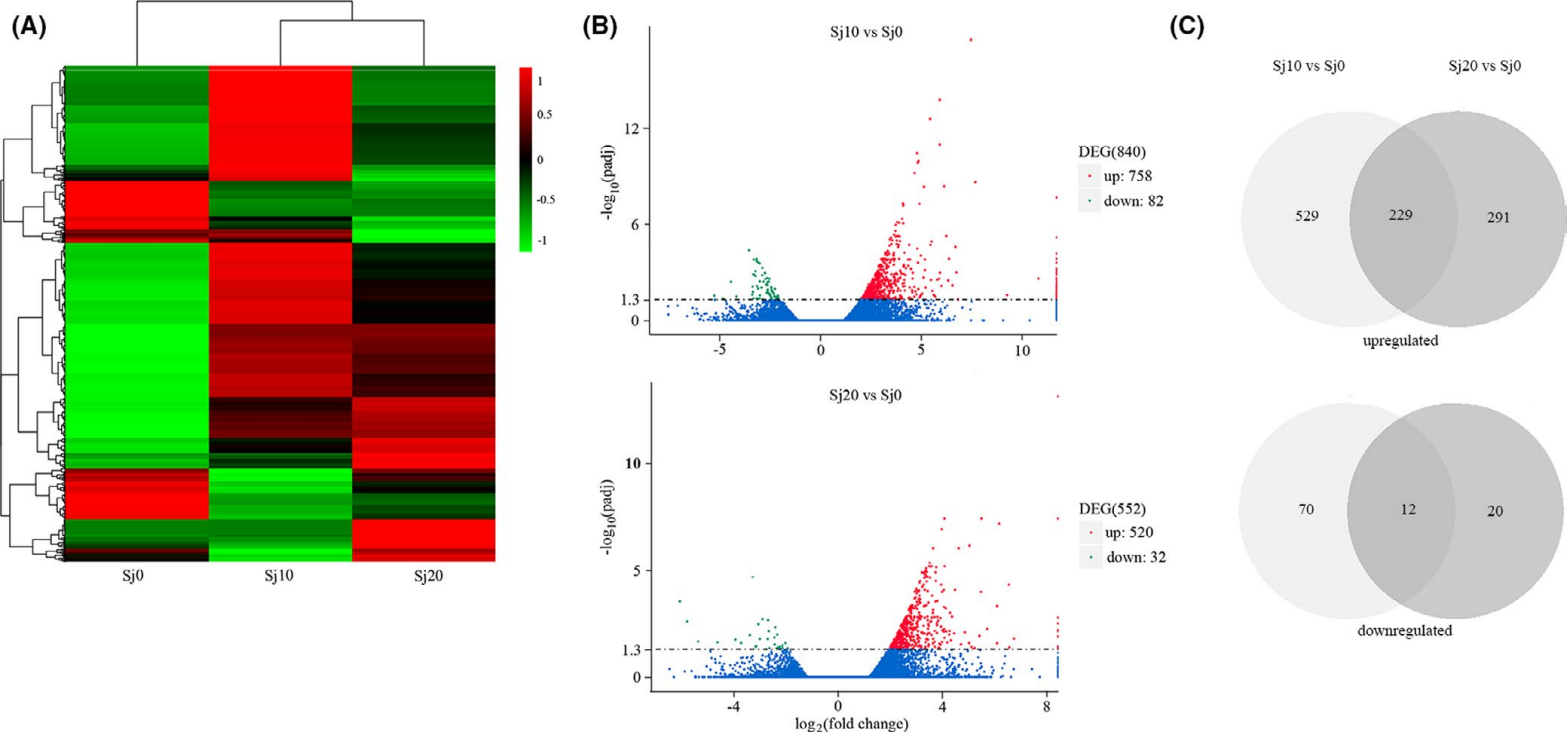
Expression profiles of DEGs between control and infected groups at different time points. (A) Hierarchical clustering of DEGs in livers of *M. fortis* infected with *S. japonicum* between control and infected groups at different time points based on log10‐transformed expression values (RPKM). The colour scale indicates the gene expression level: red colour represents increased transcript abundance and blue colour represents decreased transcript abundance; (B) Volcano plots of DEGs between the infected for 10 days group with control group, and the infected for 20 days group with control group; (C) Venn diagram described the common differential expressed genes at different time points of *M. fortis* infected with *S. japonicum*. Overview of the DEGs for each comparison samples. Sj10 stands for the infected for 10 days group; Sj20 stands for the infected for 20 days group; Sj0 stands for control group

To further analyse the expression profiles of the DEGs in *M. fortis* infected with *S. japonicum*, we compared the RNAseq data from the *M. fortis* infected with *S. japonicum* at different time points (Figure 5A). Our results showed that there were 840 DEGs including 758 up‐regulated and 82 down‐regulated DEGs between the infected for 10 days group and the control group, while 552 DEGs including 520 up‐regulated and 32 down‐regulated DEGs were found between the infected for 20 days group and the control group (Figure 5B). To be noted, 229 up‐regulated DEGs showed same expression patterns between the two groups infected at different time points, including JAK2 and STAT1 (Figure 5C), while 12 down‐regulated DEGs including Cyp2b28‐ps and PCCB showed same expression patterns between the two groups infected at different time points (Figure 5C).

**FIGURE 6:**
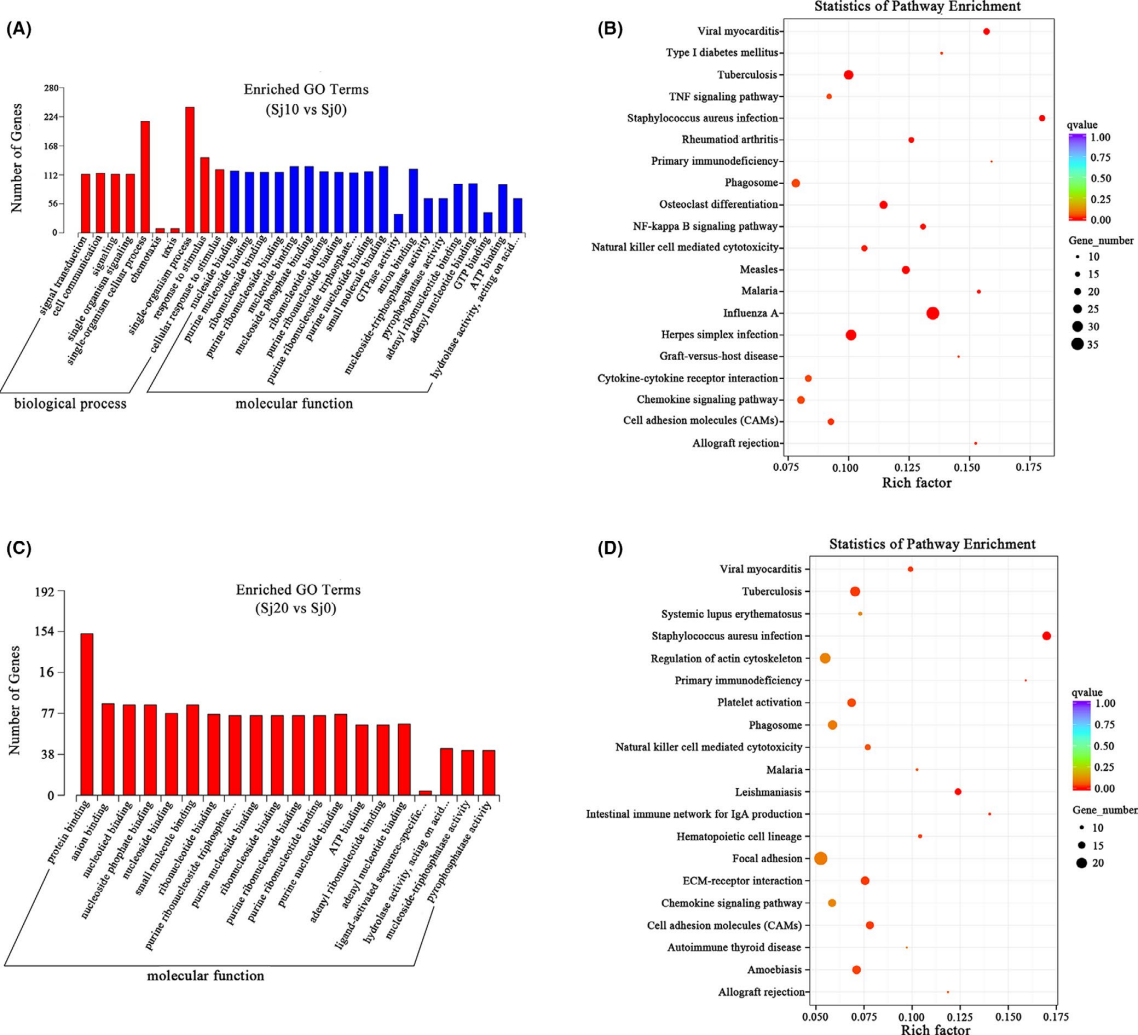
GO and KEGG enrichment analysis of the DEGs. (A) Histograms of the enriched GO terms of *M. fortis* infected with *S. japonicum* for 10 days; (B) Scatter plots of the top 20 enriched KEGG pathway terms of *M. fortis* infected with *S. japonicum* for 10 days; (C) Histograms of the enriched GO terms of *M. fortis* infected with *S. japonicum* for 20 days; (D) Scatter plots of the top 20 enriched KEGG pathway terms of *M. fortis* infected with *S. japonicum* for 20 days. GO enriched items were selected by *P* < .05. KEGG enriched items were measured by the rich factor, q value (q < .05) and the number of genes

GO enrichment analysis was then performed. In the biological process categories, ‘single‐organism cellular process’, ‘single‐organism process’ and ‘response to stimulus’ were the most prevalent in the infected for 10 days group (Figure 6A). As the infection time extended to 20 days, the number of DEGs decreased. The DEGs genes identified in this group were enriched in the category of ‘molecular function’. Among them, ‘protein binding’, ‘nucleotide binding’ and ‘nucleoside phosphate binding’ were the most prevalent in the infected for 20 days group (Figure 6C).

Moreover, all DEGs were mapped to the reference pathway in the KEGG database to identify the response pathways involved in the infection. However, only a few genes could be annotated to the KEGG pathways in the infected for 10 days group. Among them, most DEGs were involved in the categories of ‘human diseases’, ‘environmental information processing’ and ‘cellular process’. The ‘infectious diseases’, ‘immune diseases’ and ‘phagosome’ were highly enriched in these categories (Figure 6B). To be noted, ‘JAK/STAT signaling pathway’, which is related to regulate immune response was also enriched. Less DEGs were detected in the infected for 20 days group. The enriched categories were also related to ‘human diseases’, ‘organismal systems’ and ‘environmental information processing’ (Figure 6D). The highly enriched pathways were the ‘infectious diseases’, ‘immune diseases’, ‘ECM‐receptor interaction’ and ‘cell adhesion molecules (CAMs)’.

### 3.4 Immune‐function related DEGs

**FIGURE 7:**
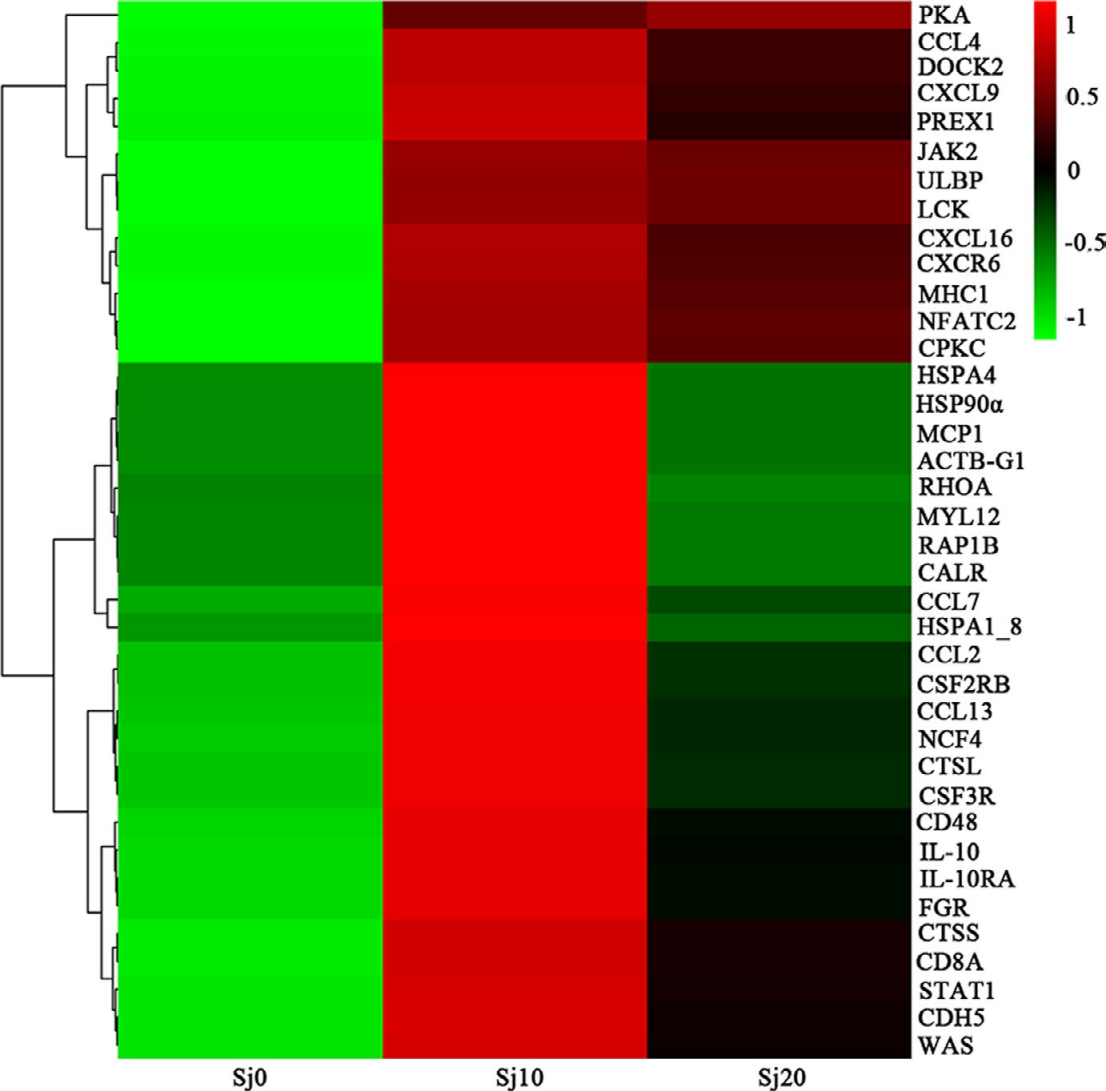
Hierarchical clustering of DEGs in immune system‐related pathway based on the log ratio fold change data. The colour scale indicates the gene expression level: red colour represents increased transcript abundance, and blue colour represents decreased transcript abundance. Sj10 stands for the infected for 10 days group; Sj20 stands for the infected for 20 days group; Sj0 stands for control group

KEGG analysis showed that up‐regulated DEGs were present in five ‘immune system’ pathways, most of which were significantly enriched (corrected *P*‐value <.05). Moreover, GO analysis also confirmed these results by showing that 18 DEGs were mapped in the ‘immune system process’, 16 DEGs in the ‘immune response’, 1 in the ‘immune system development’ and 1 in the ‘regulation of immune system process’ (Table 5). Therefore, the immune response system was active in *M. fortis* when infected by *S. japonicum*. The KEGG enrichment analysis of DEGs also suggested that ‘immune system’ related pathway, ‘natural killer cell mediated cytotoxicity’, ‘chemokine signaling pathway’, ‘antigen processing and presentation’, ‘leukocyte transendothelial migration’ and the ‘JAK/STAT signaling pathway’ were profoundly enriched. The mRNA expression level of PKA continuous increase, IL‐10, JAK2, STAT1, HSP90α were increased after infected with *S. japonicum* for 10 days in *M*. *fortis* (Figure 7, Table 6).

**TABLE 5:** Immune‐related pathways in KEGG and GO Terms

Data base	Pathway	Up	Down	DEG list	Corrected_*P*‐value
GO	immune system process	18	0	542	.0763
	immune response	16	0	542	.0938
	immune system development	1	0	542	1
	regulation of immune system process	1	0	542	1
KEGG	Intestinal immune network for IgA production	8	0	57	.0210
	Hematopoietic cell lineage	10	0	96	.0241
	Platelet activation	17	0	248	.0369
	Natural killer cell mediated cytotoxicity	13	0	169	.0450
	Chemokine signalling pathway	16	0	274	.1100

**TABLE 6:** Immune system‐related signalling pathway of DEGs in KEGG enrichement

Immune system‐related signalling pathway	Sample number	Background number	Corrected *P*‐value	Related DEGs
Natural killer cell mediated cytotoxicity	18	169	.0061	KLRC,RAC2,ULBP,ICAM1,MHC1,LCK,FCER1G,ITGAL,VAV,SYK,CPKC,MHC1,ITGB2,LCP2 GZMB,NFATC2, NFAT1, NFATP, FCGR3, CD16, CD48, BCM1
Chemokine signalling pathway	22	274	.0317	ROCK1,CCL15_23,WAS,DOCK2,SRC,FGR,SRC2,PREX1,RHOA,VAV,JAK2,CXCL9,RAC2,CXCL16,RAP1B,CCL13,CCL2,MCP1,PKA,CCL2,MCP1,STAT1,CXCR6,CCL2, MCP1,CCL4
Antigen processing and presentation	13	130	0.0357	KLRC,CTSL,htpG, HSP90A，CD8A,CTSS,MHC1,HSPA4，MHC1,CALR MHC2,MHC2，HSPA1_8,CIITA
Leukocyte transendothelial migration	17	201	0.0423	CDH5,RAC2,ICAM1,CPKC， RHOA,ITGAL,ROCK1,RAP1B,MYL12, ACTB_G1,ITGB2，VIL2,MSN,VAV,NCF4,P40, PHOX
Jak‐STAT signalling pathway	9	153	.7075	IL10, CSIF, OSMR,CSF2RB, IL3RB,JAK2,SOCS3,CIS3，STAT1，IL10RA, SOCS1, JAB,CSF3R

Note

The selected pathway were identified using hypergeometric test method, Benjamini and Hochberg method. The sample number is the DEGs numbers of *M. fortis* infected with *S. japonicum* in immune system‐related signalling pathway in KEGG database. The background number is the related gene numbers in immune system‐related signalling pathway in KEGG database.

### 3.5 qRT‐PCR verification of the RNA‐Seq data

**FIGURE 8:**
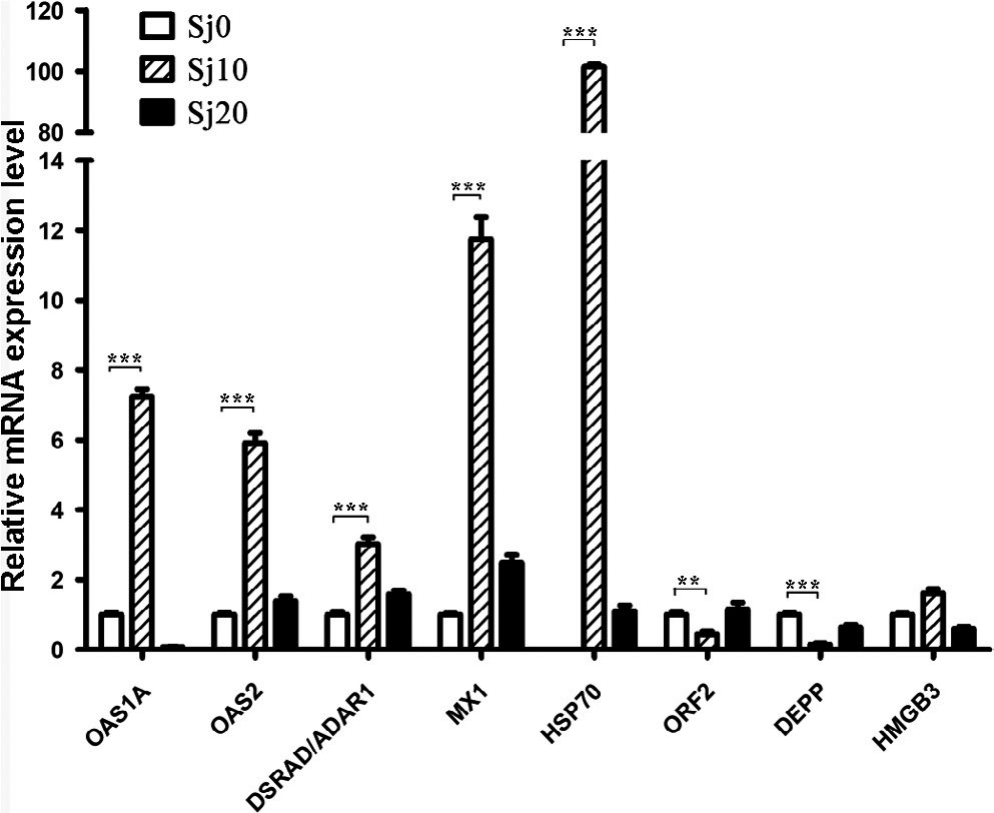
Verification the DEGs of RNA‐seq by qRT‐PCR. The relative expression levels of selected genes were detected by qRT‐PCR for the uninfected or post‐infected *M. fortis*. Sj10 and Sj20 stand for the infected for 10 days and 20 days group, respectively; Sj0 stands for control group. Error bars indicate standard deviation. *P < .05; *P < .01, ***P < .001

Eight DEGs were randomly selected just to verify the results of the RNA‐Seq analysis by qRT‐PCR. The results from the tested genes displayed significant differential expression among the control and infected groups at different time points, which was similar to the DEGs pattern obtained from RNAseq (Figure 8). The OAS1A, OAS2, DSRAD/ADAR1, MX1 and HSP70 were up‐regulated whereas ORF2 and DEEP showed low abundance in the post‐infected for 10 days group. The results from the qRT‐PCR data matched with the RNA‐Seq data, indicating that the RNA‐Seq data were reliable.

### 3.6 Infection‐induced IL‐10 and JAK/STAT signalling pathway

**FIGURE 9:**
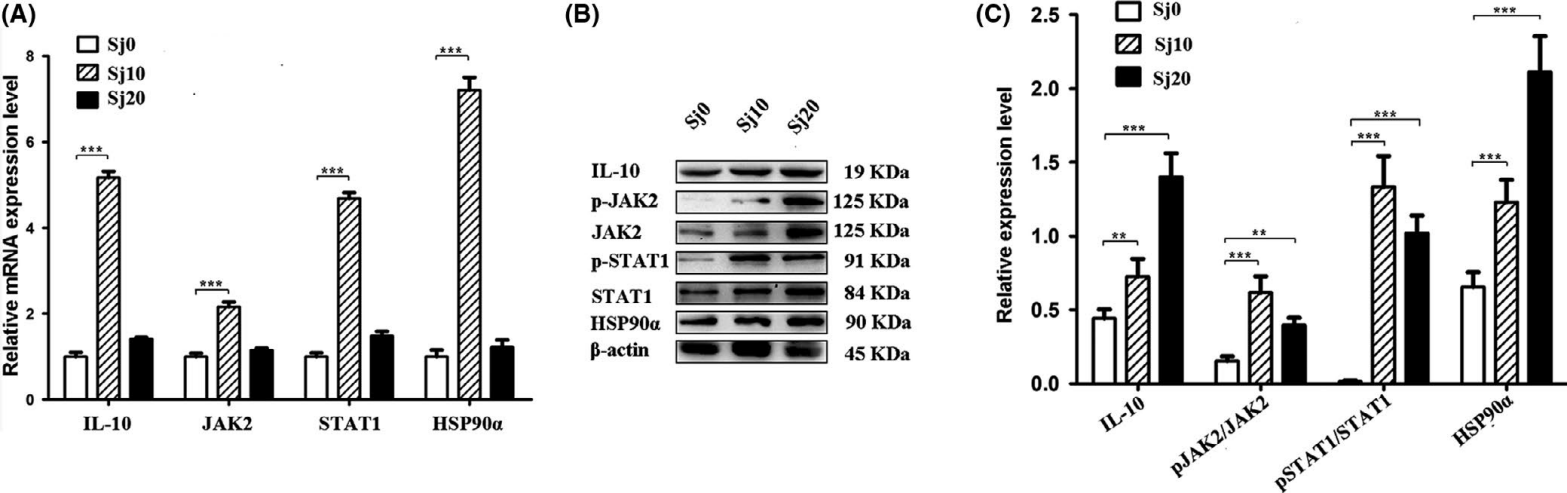
Detection of relative expression level of IL‐10, JAK2, STAT1 and HSP90*α* in the liver of *M. fortis* by qRT‐PCR and western blot. (A) Expression of IL‐10, JAK2, STAT1 and HSP90*α* at the mRNA level of *M. fortis* infected with *S. japonicum* for 10 days and 20 days; (B) Expression of IL‐10, JAK2, p‐JAK2, STAT1, p‐STAT1 and HSP90*α* at the protein level of *M. fortis* infected with *S. japonicum* for 10 days and 20 days. Sj10 and Sj20 stands for the infected for 10 days and 20 days group, respectively; Sj0 stands for control group. Error bars indicate standard deviation, ***P < .001, **P < .01

HSP90*α* was profoundly increased after infected with *S. japonicum* by performing GO and KEGG analysis of the DEGs. In order to verify our hypothesis, we detected the expression of IL‐10, JAK2, STAT1 and Hsp90*α* by qRT‐PCR and Western blot. The results showed that the messenger RNA expression levels of IL‐10, JAK2, STAT1 and HSP90*α* were increased after infected with *S. japonicum* for 10 days in *M. fortis*, and their expressions were gradually decreased after infected for 20 days (Figure 9A). The results from Western blot might indicate that after infected with *S. japonicum* for 10 days and 20 days, the HSP90α expression level was elevated by phosphorylation of JAK/STAT signalling cascade after the increase in IL‐10, as shown by the significantly increase expression level of IL‐10, p‐JAK2 (Tyr119)/JAK2, p‐STAT1 (Ser727)/STAT1 and HSP90α (Figure 9B). It suggested that after infection with *S. japonicum*, the JAK/STAT signalling pathway was activated with the increased expression of IL‐10.

## 4 DISCUSSION

In the current study, we utilized RNA‐Seq to obtain the transcriptome profile of *M. fortis* infected with *S. japonicum* for 10 days and 20 days. By performing GO and KEGG analysis of the DEGs, we found that the JAK/STAT signalling pathway was one of the most prominent enriched pathways involved in the pathological process of *M. fortis* infected with *S. japonicum* at different time points, and JAK2 and STAT1 were among the up‐regulated DEGs that showed same expression patterns between the two groups infected at different time points. Moreover, IL‐10 is a prototypic anti‐inflammatory cytokine, and enhanced whole‐blood IL‐10 secretion was detected in response to cercarial excretory/secretory products after schistosome infection.^25^ The IL‐10‐JAK‐STAT axis has been suggested to participate in the pathogenesis of microbe infection.^26^

In our previous study, Mf‐HSP90*α*, KPNA2 and albumin were found to be the resistance‐associated proteins of *M. fortis*.^17^, ^18^, ^19^ In order to explore the resistance mechanism of Mf‐HSP90*α*, KPNA2 and ALB, RNA‐Seq was performed to obtain the transcriptome profile of the liver of *M. fortis* infected with *S. japonicum* for 10 days and 20 days, and the uninfected *S. japonicum* were used as control. Mf‐HSP90*α* was profoundly increased after infected with *S. japonicum* by performing GO and KEGG analysis of the DEGs, while there were no significant changes of albumin and KPNA2. These results prompt us to explore the mechanism of Mf‐HSP90*α*’s resistance effect to *S. japonicum* infection. Herein, by analysing the transcriptome profile of the *M. fortis* infected with *S. japonicum* at different time points and mapping the DEGs to GO and KEGG, we propose that the anti‐schistosome effect of Mf‐HSP90*α* is driven by activated JAK2/STAT1 pathway after an increase in IL‐10 level.

IL‐10 is a prototypic anti‐inflammatory cytokine that is produced in response to a multitude of pathogens and plays an important role in controlling acute granulomatous inflammation.^27^, ^28^ It is also reported that IL‐10 is able to activate promoter of HSP90*α* directly.^29^ The enhanced whole‐blood IL‐10 secretion was detected after schistosome infection, including infection with *S. japonicum*.^25^, ^30^ The elevated IL‐10 level is able to activate JAK/STAT signalling cascade, mostly through JAK1/STAT3.^26^ The p‐JAK2/JAK2 and p‐STAT1/STAT1 ratio reached highest level at 10 days post‐infection and decreased at 20 days post‐infection. IL‐10 is able to activate the JAK2/STAT1 pathway (Figure S2) and activate promoter of HSP90*α* directly.^29^ Moreover, HSP90 is a direct target of STAT1.^31^ These results and clues indicated that the IL‐10 activate HSP90*α* through JAK2/STAT1 pathway. The IL‐10‐JAK2/STAT1‐HSP90*α* axis is likely to be responsible for the anti‐schistosome effect of Mf‐HSP90*α*. Once activated by IL‐10, JAK2 phosphorylate tyrosine residues of both the receptors and the STAT1. Phosphorylated STAT1 proteins dimerize and translocate into the nucleus, where they function as transcriptional activators for HSP90*α*, leading to the enhancement of killing schistosome‐killing effect of HSP90*α*.

Our current study mainly focused on finding the up‐stream signalling cascade responsible for the anti‐schistosome effect of Mf‐HSP90*α*. We obtained the transcriptome profile of *M. fortis* infected with *S. japonicum* at different time points, and by employing bioinformatic tools, we found the signalling pathway that is likely to be responsible for the activation of Mf‐HSP90*α*. Our study not only broadens our knowledge of the mechanism of Mf‐HSP90*α*'s resistance effect on *S. japonicum,* but we also propose a novel therapeutic strategy for targeting *S. japonicum*. However, whether the elevated IL‐10 and activated JAK2/STAT1 pathway have other downstream effectors that can exert anti‐schistosome effects merits further investigation (Figure S2). For instance, IL‐10 was able to block the development of resistance to re‐infection with *S. mansoni* by increasing in IL‐10‐producing CD4(+)CD44(+)CD25(+)GITR(+) lymphocytes.^32^ Moreover, whether the anti‐schistosome effect of Mf‐HSP90*α* is driven by other signalling pathway is still one of our main concerns.

There were some limitations of this study. The number of the animals used in this study has not been calculated.

In conclusion, we showed that the anti‐schistosome effect of Mf‐HSP90*α* is likely to be activated by the phosphorylated JAK2/STAT1 pathway followed by increased IL‐10 secretion. Our results suggested that the IL‐10‐JAK2/STAT1‐Mf‐HSP90*α* axis may be responsible for resistance effect of *M. fortis* to *S. japonicum* infection.

## CONFLICT OF INTEREST

The authors declare no potential conflicts of interest.

## AUTHOR CONTRIBUTIONS

WXH and JPH conceived and wrote the manuscript. DHX, SQL, JMS, YPW designed and performed the experiments. DHX, SQL, KLW, YJY, WXH and ​JPH evaluated and analysed the results.

## Supporting information

Figure S1

Figure S2

Table S1‐S3

## Data Availability

The RNA‐Seq sequencing data have been deposited into the Sequence Read Archive(SRA) database (http://www.ncbi.nlm.nih.gov/Traces/sra/) under accession number PRJNA722951. All other relevant data are within the paper and its supporting information files.
