# Supplementary figures and images for: Transcriptional profiling of Microtus fortis responses to S. japonicum: New sight into Mf‐Hsp90α resistance mechanism
Source: Parasite Immunol. 2021 Jun 9;43(8):e12842. doi: 10.1111/pim.12842 (PMC8365665; doi:10.1111/pim.12842)

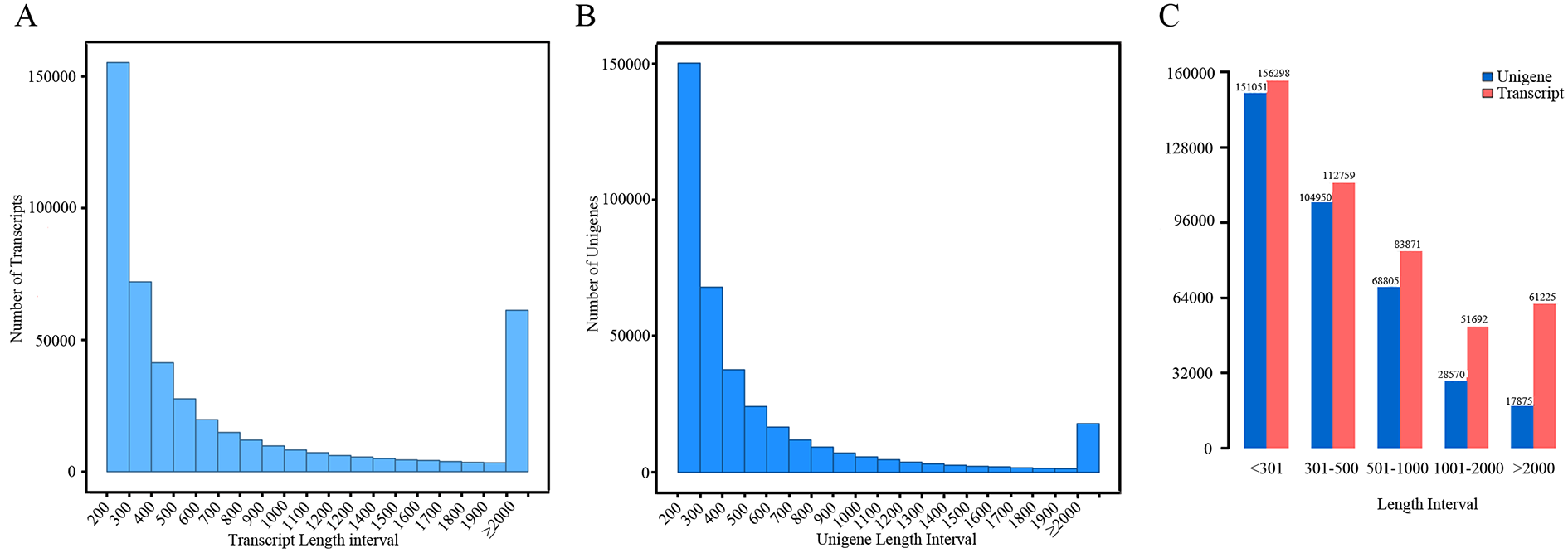

Supplement: Supplementary file 1 — Figure S1 [file PIM-43-e12842-s001.tif]

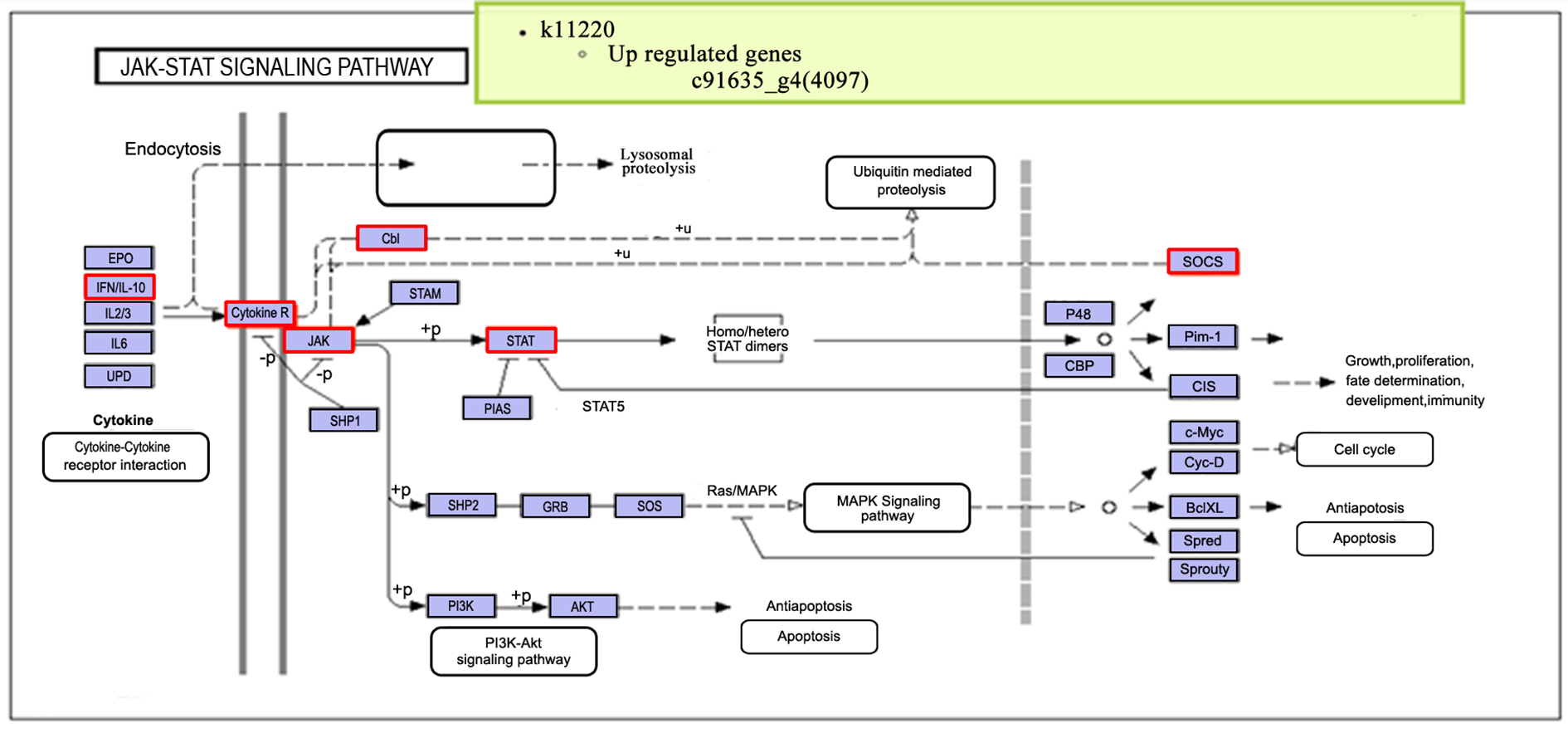

Supplement: Supplementary file 2 — Figure S2 [file PIM-43-e12842-s003.tif]
