# Supplementary material for: Transcriptional profiling of Microtus fortis responses to S. japonicum: New sight into Mf‐Hsp90α resistance mechanism
Source: Parasite Immunol. 2021 Jun 9;43(8):e12842. doi: 10.1111/pim.12842 (PMC8365665; doi:10.1111/pim.12842)
Supplement: Supplementary file 3 — Table S1‐S3 [file PIM-43-e12842-s002.docx]

**Table S1 The GO annotation of assembled genes**

| **GO ID** | **GO Term (Lev2)** | **GO Term (Lev1)** | **Gene Number** |
| --- | --- | --- | --- |
| GO:0045182 | binding | Molecular Function | 19982 |
| GO:0016530 | catalytic activity | Molecular Function | 14504 |
| GO:0030545 | transporter activity | Molecular Function | 2654 |
| GO:0005085 | structural molecule activity | Molecular Function | 1392 |
| GO:0016209 | nucleic acid binding transcription factor activity | Molecular Function | 1335 |
| GO:0000988 | molecular transducer activity | Molecular Function | 1137 |
| GO:0016247 | enzyme regulator activity | Molecular Function | 914 |
| GO:0004872 | receptor activity | Molecular Function | 898 |
| GO:0030234 | channel regulator activity | Molecular Function | 448 |
| GO:0060089 | protein binding transcription factor activity | Molecular Function | 405 |
| GO:0001071 | antioxidant activity | Molecular Function | 124 |
| GO:0005198 | guanyl-nucleotide exchange factor activity | Molecular Function | 104 |
| GO:0005215 | receptor regulator activity | Molecular Function | 70 |
| GO:0003824 | metallochaperone activity | Molecular Function | 21 |
| GO:0005488 | translation regulator activity | Molecular Function | 2 |
| GO:0005623 | cell | Cellular Component | 13258 |
| GO:0044464 | cell part | Cellular Component | 13257 |
| GO:0043226 | organelle | Cellular Component | 8617 |
| GO:0032991 | macromolecular complex | Cellular Component | 8295 |
| GO:0016020 | membrane | Cellular Component | 7368 |
| GO:0044425 | membrane part | Cellular Component | 6807 |
| GO:0044422 | organelle part | Cellular Component | 4443 |
| GO:0031974 | membrane-enclosed lumen | Cellular Component | 1388 |
| GO:0044423 | virion part | Cellular Component | 1224 |
| GO:0019012 | virion | Cellular Component | 1224 |
| GO:0005576 | extracellular region | Cellular Component | 1032 |
| GO:0044421 | extracellular region part | Cellular Component | 1024 |
| GO:0031012 | extracellular matrix | Cellular Component | 300 |
| GO:0044420 | extracellular matrix part | Cellular Component | 123 |
| GO:0030054 | cell junction | Cellular Component | 69 |
| GO:0045202 | synapse | Cellular Component | 39 |
| GO:0044456 | synapse part | Cellular Component | 34 |
| GO:0009295 | nucleoid | Cellular Component | 6 |
| GO:0009987 | cellular process | Biological Process | 22412 |
| GO:0008152 | metabolic process | Biological Process | 19332 |
| GO:0044699 | single-organism process | Biological Process | 17122 |
| GO:0065007 | biological regulation | Biological Process | 9477 |
| GO:0050789 | regulation of biological process | Biological Process | 9063 |
| GO:0050896 | response to stimulus | Biological Process | 7016 |
| GO:0051179 | localization | Biological Process | 6075 |
| GO:0023052 | signaling | Biological Process | 4687 |
| GO:0071840 | cellular component organization or biogenesis | Biological Process | 3915 |
| GO:0051704 | multi-organism process | Biological Process | 3257 |
| GO:0032501 | multicellular organismal process | Biological Process | 1362 |
| GO:0032502 | developmental process | Biological Process | 1060 |
| GO:0022610 | biological adhesion | Biological Process | 726 |
| GO:0048518 | positive regulation of biological process | Biological Process | 617 |
| GO:0048519 | negative regulation of biological process | Biological Process | 596 |
| GO:0002376 | immune system process | Biological Process | 534 |
| GO:0000003 | reproduction | Biological Process | 464 |
| GO:0040011 | locomotion | Biological Process | 446 |
| GO:0022414 | reproductive process | Biological Process | 353 |
| GO:0040007 | growth | Biological Process | 114 |
| GO:0001906 | cell killing | Biological Process | 72 |
| GO:0048511 | rhythmic process | Biological Process | 36 |
| GO:0044848 | biological phase | Biological Process | 21 |
| GO:0046879 | hormone secretion | Biological Process | 9 |

**Table S2 The KOG annotation of assembled genes**

| Category | Description | Number | Ratio (%) |
| --- | --- | --- | --- |
| A | RNA processing and modification | 731 | 4.81 |
| B | Chromatin structure and dynamics | 290 | 1.91 |
| C | Energy production and conversion | 523 | 3.44 |
| D | Cell cycle control, cell division, chromosome partitioning | 449 | 2.96 |
| E | Amino acid transport and metabolism | 437 | 2.88 |
| F | Nucleotide transport and metabolism | 190 | 1.25 |
| G | Carbohydrate transport and metabolism | 434 | 2.86 |
| H | Coenzyme transport and metabolism | 142 | 0.94 |
| I | Lipid transport and metabolism | 529 | 3.48 |
| J | Translation, ribosomal structure and biogenesis | 982 | 6.47 |
| K | Transcription | 891 | 5.87 |
| L | Replication, recombination and repair | 343 | 2.26 |
| M | Cell wall/membrane/envelope biogenesis | 109 | 0.72 |
| N | Cell motility | 27 | 0.18 |
| O | Posttranslational modification, protein turnover, chaperones | 1646 | 10.84 |
| P | Inorganic ion transport and metabolism | 509 | 3.35 |
| Q | Secondary metabolites biosynthesis, transport and catabolism | 234 | 1.54 |
| R | General function prediction only | 2629 | 17.31 |
| S | Function unknown | 885 | 5.83 |
| T | Signal transduction mechanisms | 2749 | 18.10 |
| U | Intracellular trafficking, secretion, and vesicular transport | 1032 | 6.80 |
| V | Defense mechanisms | 184 | 1.21 |
| W | Extracellular structures | 306 | 2.02 |
| X | Unamed protein | 4 | 0.03 |
| Y | Nuclear structure | 105 | 0.69 |
| Z | Cytoskeleton | 863 | 5.68 |

**Table S3 The KEGG annotation of assembled genes**

| **Pathway Hierarchy1** | **Pathway Hierarchy2** | **Number** |
| --- | --- | --- |
| Cellular Processes | Cell growth and death | 571 |
| Cellular Processes | Cell motility | 365 |
| Cellular Processes | Cellular commiunity | 859 |
| Cellular Processes | Transport and catabolism | 1050 |
| Environmental Information Processing | Membrane transport | 108 |
| Environmental Information Processing | Signal transduction | 2574 |
| Environmental Information Processing | Signaling molecules and interaction | 887 |
| Genetic Information Processing | Folding, sorting and degradation | 958 |
| Genetic Information Processing | Replication and repair | 281 |
| Genetic Information Processing | Transcription | 452 |
| Genetic Information Processing | Translation | 1157 |
| Metabolism | Amino acid metabolism | 565 |
| Metabolism | Biosynthesis of other secondary metabolites | 97 |
| Metabolism | Carbohydrate metabolism | 651 |
| Metabolism | Energy metabolism | 536 |
| Metabolism | Glycan biosynthesis and metabolism | 328 |
| Metabolism | Lipid metabolism | 633 |
| Metabolism | Metabolism of cofactors and vitamins | 351 |
| Metabolism | Metabolism of other amino acids | 232 |
| Metabolism | Metabolism of terpenoids and polyketides | 66 |
| Metabolism | Nucleotide metabolism | 349 |
| Metabolism | Overview | 424 |
| Metabolism | Xenobiotics biodegradation and metabolism | 294 |
| Organismal Systems | Circulatory system | 457 |
| Organismal Systems | Development | 445 |
| Organismal Systems | Digestive system | 670 |
| Organismal Systems | Endocrine system | 1150 |
| Organismal Systems | Environmental adaptation | 289 |
| Organismal Systems | Excretory system | 302 |
| Organismal Systems | Immune system | 1268 |
| Organismal Systems | Nervous system | 781 |
| Organismal Systems | Sensory system | 317 |
